# 329. Vancomycin area under the concentration-time curve monitoring from Bayesian modeling in outpatient parenteral antimicrobial therapy

**DOI:** 10.1093/ofid/ofad500.400

**Published:** 2023-11-27

**Authors:** Eric M Gillett, Muneerah M Aleissa, Jeffrey C Pearson, David W Kubiak, Brandon Dionne, Brian T Chan

**Affiliations:** Brigham and Women's Hospital, Cambridge , Massachusetts; Brigham and Women's Hospital, Cambridge , Massachusetts; Brigham and Women's Hospital, Cambridge , Massachusetts; Brigham & Women's Hospital, Boston, Massachusetts; Brigham and Women's Hospital, Cambridge , Massachusetts; Brigham and Women's Hospital, Cambridge , Massachusetts

## Abstract

**Background:**

Current vancomycin monitoring guidelines recommend area under the concentration-time curve to minimum inhibitory concentration (AUC/MIC) monitoring for patients with serious MRSA infections. Most AUC monitoring studies have included hospitalized patients only; however, many patients complete vancomycin therapy in the outpatient setting. While traditional two-level AUC calculations are logistically challenging for outpatients, Bayesian modeling software can allow for single level AUC estimations. There are sparse data on the feasibility, safety, and efficacy of vancomycin AUC monitoring in OPAT.

**Methods:**

We conducted a single-center, quasi-experimental, interrupted time series study of adult patients enrolled in the OPAT program at our institution for vancomycin management. Our institution implemented a pharmacist-driven vancomycin monitoring program using AUC targets from September 8^th^, 2019 to February 25^th^, 2020, and again from September 25^th^, 2022 to March 16^th^, 2023. Patients enrolled in the monitoring program underwent vancomycin monitoring using an AUC goal of 400-600 mg*hr/L, estimated using Bayesian modeling. A cohort comprising all patients enrolled in the OPAT program from July 4^th^, 2021 through August 27^th^, 2022 for trough only vancomycin monitoring was used for comparison. Patients were excluded from analysis if they were undergoing renal replacement therapy at baseline, enrolled in OPAT for ≤ 72 hours, or did not have vancomycin levels drawn for ≥ 14 days. The primary outcome was nephrotoxicity defined as a serum creatinine increase by ≥ 0.5 mg/dL or ≥ 50% during outpatient vancomycin therapy.

**Results:**

There were 63 patients in the AUC arm, and 60 patients in the trough-only arm. The incidence of nephrotoxicity was lower in the AUC cohort (6.3% vs 23.3%, p=0.01). There was no difference in other vancomycin-related adverse event rates (1.6% vs 10.0%, p=0.06). Vancomycin discontinuation due to adverse event was lower in the AUC arm (6.3% vs 20.0%, p=0.03).

**Conclusion:**

Patients undergoing AUC monitoring were significantly less likely to develop nephrotoxicity during outpatient vancomycin treatment. Bayesian modeling represents a viable and safe option for vancomycin AUC monitoring in OPAT.

**Disclosures:**

**David W. Kubiak, PharmD, BCPS, BCIDP, FIDSA**, Astellas Pharma, Inc.: Advisor/Consultant|AVIR Pharma Inc: Advisor/Consultant|Cidara Therapeutics: Advisor/Consultant|Shionogi Inc: Grant/Research Support

